# The Development and Initial Validation of the REST Questionnaire: A Multidimensional Tool for Assessing Fatigue in Individuals with and Without a History of Cancer

**DOI:** 10.3390/diseases13010015

**Published:** 2025-01-15

**Authors:** Giuseppe Di Lorenzo, Carlo Buonerba, Raffaele Baio, Eleonora Monteleone, Francesco Passaro, Antonio Tufano, Vittorino Montanaro, Vittorio Riccio, Ilaria Gallo, Francesca Cappuccio, Federica Fortino, Anna Buonocore, Federica Monaco, Antonio Verde, Anna Rita Amato, Oriana Strianese, Ferdinando Costabile, Luca Scafuri

**Affiliations:** 1 Oncology Unit, “Andrea Tortora” Hospital, ASL Salerno, 84016 Pagani, Italy; giuseppe.dilorenzo@unicamillus.org (G.D.L.); cappuccio.francesca@tiscali.it (F.C.); xt.fortinof@aslsalerno.it (F.F.); antonioverde93@gmail.com (A.V.); annarita.amato93@gmail.com (A.R.A.); xt.strianeseo@aslsalerno.it (O.S.); ferdicostabile@gmail.com (F.C.); lucaluca@hotmail.it (L.S.); 2 Associazione O.R.A. ETS-Oncology Research Assistance, 84134 Salerno, Italy; 3 Department of Medicine, UniCamillus-Saint Camillus International University of Health Sciences, 00131 Rome, Italy; 4 Department of Urology, Umberto I, Nocera Inferiore, 84014 Salerno, Italy; dott.rbaio@gmail.com; 5 Department of Urology, Istituito Nazionale Tumori IRCCS Fondazione G. Pascale, 80131 Napoli, Italy; eleonora.m@live.com (E.M.); francescopassaro1996@gmail.com (F.P.); antonio.tufano91@gmail.com (A.T.); 6 Urology Unit, San Leonardo Hospital, Castellammare Di Stabia, 80053 Naples, Italy; vittorino.montanaro@alice.it; 7 Ospedale S. Maria della Pietà, 80026 Casoria, Italy; vittorioriccio1990@gmail.com; 8 Primary Care Department, ASL Salerno (SA), 84016 Pagani, Italy; ilariag.10@libero.it (I.G.); anna.buonocore1962@gmail.com (A.B.); 9 Department of Anesthesia, San Paolo Hospital ASL Napoli 1 Centro, 80125 Naples, Italy; monacofederica@gmail.com

**Keywords:** fatigue assessment, REST questionnaire, psychometrics, validation study, public health, well-being, chronic conditions

## Abstract

Background: Fatigue is a prevalent and complex condition with significant impacts on well-being. Existing fatigue assessments often lack comprehensiveness or practicality for general population studies. Methods: This study validated the REST Questionnaire, a novel fatigue assessment tool, in a sample of 268 adults. Psychometric properties, including internal consistency and construct validity, were evaluated. REST scores were correlated with WHO-5 well-being, BMI, self-rated health, and chronic conditions. Exploratory factor analysis identified underlying dimensions of fatigue. Results: The REST Questionnaire demonstrated excellent internal consistency (Cronbach’s alpha = 0.918) and construct validity. Higher fatigue scores were associated with lower well-being, female gender, and the presence of certain chronic conditions (cancer, kidney stones, gastric ulcers). Two distinct fatigue dimensions, “physical fatigue and functional impacts” and “emotional and social consequences”, were identified. Conclusions: The REST Questionnaire is a reliable and valid tool for assessing fatigue in the general population. Its multidimensional framework and sensitivity to comorbidities offer valuable insights for research and public health applications, with the potential to inform targeted interventions aimed at improving well-being.

## 1. Introduction

Fatigue is a pervasive and multifaceted condition that significantly affects physical, emotional, and social well-being. It is often characterized by feelings of exhaustion, reduced motivation, and impaired functioning, with implications for both individual health and societal productivity [1]. Despite its widespread impact, fatigue remains challenging to assess due to its subjective nature and the broad spectrum of symptoms it encompasses [2]. Existing fatigue questionnaires, such as the Fatigue Severity Scale (FSS) and the Chalder Fatigue Scale (CFS), have been instrumental in advancing our understanding of fatigue, particularly in clinical settings and among populations with specific health conditions [3,4]. However, these tools often focus on a narrow set of symptoms or fail to fully capture the broader psychosocial and functional impacts of fatigue, limiting their applicability to general population research.

Furthermore, questionnaires like the FSS and CFS [5] lack sensitivity to lifestyle factors such as diet, physical activity, and sleep quality, which are increasingly recognized as critical determinants of fatigue. Tools like the Multidimensional Fatigue Inventory (MFI) offer a more comprehensive approach but are often lengthy and cumbersome for use in large-scale studies [6]. This gap underscores the need for a new instrument that not only measures fatigue across its multiple dimensions but also aligns with the practical requirements of public health research and population-based studies.

The REST Questionnaire was developed by two of the authors (GDL and CB) to address these limitations by offering a concise yet comprehensive tool designed specifically for the general population. Unlike many existing fatigue scales, the REST Questionnaire incorporates a holistic perspective, capturing not only the physical symptoms of fatigue but also its emotional and functional impacts. It also emphasizes the role of modifiable lifestyle factors, making it particularly suited for research exploring interventions to reduce fatigue and improve well-being. This innovation lies at the intersection of precision and practicality, combining robust psychometric properties with ease of use in diverse settings.

The present study aims to validate the REST Questionnaire by examining its reliability, construct validity, and utility in differentiating fatigue levels within the general population. By correlating REST scores with key health indicators, including the WHO-5 Well-Being Index, body mass index (BMI), self-rated health, and chronic medical conditions, this study seeks to position the REST Questionnaire as a pivotal tool for both research and public health applications. This validation effort is being conducted within the framework of the ongoing PREVES-ENERGY survey, a cross-sectional observational study investigating lifestyle factors associated with fatigue. The REST Questionnaire was included as a secondary objective in the PREVES-ENERGY survey to leverage its robust dataset and participant diversity for comprehensive validation. By integrating the REST Questionnaire into this broader study, the research benefits from access to a wide range of variables and a substantial participant pool, enhancing the reliability and generalizability of the findings. Through this effort, the REST Questionnaire aspires to bridge the gap between clinical and population-level fatigue assessment, providing new insights into fatigue’s multidimensional nature and advancing strategies for its management.

## 2. The REST Questionnaire: Conceptualization and Design

### 2.1. Development of the REST Questionnaire

The REST Questionnaire, short for Recognizing and Estimating Signs of Tiredness, was developed to address critical gaps in fatigue assessment by offering a multidimensional view that encompasses physical, emotional, functional, and behavioral domains. This tool was designed to capture fatigue not merely as a physical symptom but as a complex phenomenon influenced by individual physiology, emotional well-being, and environmental factors. To achieve this, the questionnaire includes 13 items that assess the frequency and severity of various fatigue-related experiences over the preceding 30 days. Physical symptoms are captured through questions such as “How often have you felt tired, fatigued, without energy, or exhausted?”, “How often have you experienced muscle pain?”, “How often have you suffered from headaches?”, and “How often have you felt as if your bones were aching or broken?” To evaluate functional impacts, participants are asked the following questions: “How much has fatigue prevented you from performing your usual activities (work, study, household chores, etc.)?”, “How much has fatigue limited your ability to plan the activities you want to do?”, “How much has fatigue affected your work or study life?”, and “How much has fatigue affected your social life?” Emotional dimensions are addressed through questions that explore the following: “How much has fatigue affected your emotional life and intimate relationships?”, “How frustrated have you felt because of your fatigue?”, and “How worried have you felt because of your fatigue?” Finally, behavioral responses to fatigue are assessed with items such as “How often have you felt the need to consume coffee, tea, energy drinks, or supplements to combat fatigue?” and “How often have you felt the need to seek medical help because of fatigue?”

Each item is scored on a 0-to-4 Likert scale (0 = “Not at all” to 4 = “Very much”), and the total score, ranging from 0 to 52, provides a quantitative measure of fatigue severity, with higher scores indicating greater impact. This scoring system supports individual-level assessments, helping clinicians identify specific areas where fatigue is most debilitating, while also enabling population-level comparisons across demographics and clinical groups. The questionnaire was developed iteratively, starting with a comprehensive literature review to ensure coverage of the most relevant fatigue dimensions. Items were refined through expert consultations with professionals in fatigue research, psychology, and public health to ensure clarity, comprehensiveness, and validity. Pilot testing with diverse populations further validated its cultural and situational relevance, confirming that the items resonate with a broad audience.

The REST Questionnaire is both concise and comprehensive, facilitating its use in diverse settings. Its inclusion of behavioral responses, such as the need for stimulants or medical assistance, adds depth to the understanding of how individuals cope with fatigue in real-world contexts. Additionally, by addressing anticipatory limitations (e.g., the ability to plan activities), the questionnaire sheds light on often-overlooked aspects of fatigue. By integrating physical, emotional, functional, and behavioral domains, the REST Questionnaire captures fatigue’s broad impacts on daily life, positioning it as a valuable tool for clinical and research applications.

### 2.2. Integration Within the PREVES-ENERGY Survey

Validation of the REST Questionnaire was implemented as a secondary objective of the PREVES-ENERGY survey, a cross-sectional observational study investigating lifestyle factors associated with fatigue. The ongoing PREVES-ENERGY study, approved by the Institutional Ethics Committee (CE/2024/002, 10 October 2024), aims to recruit 1000 adults aged 18 and older through healthcare professionals, excluding individuals with major illnesses under active treatment to maintain a focus on the general population.

The recruitment process involved collaborations with healthcare professionals from various specialties (e.g., general practitioners, nutritionists, and specialists in internal medicine) across different regions of Italy. These professionals were briefed on the study’s objectives and procedures and asked to disseminate an anonymous online survey link to potentially eligible patients within their practice. A statement on data protection and anonymity was presented and had to be accepted by participants to access the questionnaire. The survey was administered using Google Forms, ensuring broad accessibility. Recruitment materials emphasized the study’s focus on understanding fatigue in the general adult population. To ensure a focus on fatigue within a generally healthy population, individuals undergoing active treatment for major illnesses (e.g., active cancer treatment, recent major surgery, or acute infectious diseases) were excluded. This exclusion criterion was implemented to minimize the potential confounding effects of acute medical interventions and their associated symptoms on the assessment of fatigue. Furthermore, participants that were hospitalized or residing in assisted living facilities at the time were also excluded. However, the study did not specifically exclude individuals with a history of cancer who were not undergoing active treatment.

Participants completed an anonymous online survey capturing a range of variables. Fatigue levels were assessed using the REST Questionnaire, while overall well-being was measured through the WHO-5 Well-Being Index, a validated tool that uses five self-reported items rated on a scale from 0 to 4 to evaluate subjective well-being. Self-reported health was also measured on a 0–4 scale, with participants rating their perceived overall health status. Additionally, participants provided their weight and height, enabling the calculation of body mass index (BMI). A self-reported anamnesis was included, where individuals indicated whether they had ever been diagnosed with a list of common diseases, ensuring a comprehensive understanding of the sample’s health background. The conditions listed ranged from cardiovascular diseases (e.g., myocardial infarction, angina pectoris, stroke) and metabolic disorders (e.g., diabetes, high cholesterol) to specific malignancies and autoimmune diseases (Appendix B).

Data from at least 250 participants were required by protocol to perform a validation test of the REST Questionnaire. Exploratory factor analysis (EFA) was conducted to identify underlying dimensions of the REST Questionnaire, along with measures of internal consistency, such as Cronbach’s alpha, to validate its reliability. These analyses ensured the REST Questionnaire’s construct validity, confirming its suitability for assessing fatigue severity and its impact on daily life.

### 2.3. Objectives of the Study

The primary objective of this study is to validate the REST Questionnaire as a reliable and comprehensive tool for assessing fatigue in the general population. This validation involves a thorough examination of the psychometric properties of the questionnaire, including internal consistency, construct validity, and its ability to differentiate between fatigue levels. By correlating REST scores with key external variables—such as WHO-5 well-being scores, BMI, self-rated health, and medical conditions—this study aims to demonstrate the questionnaire’s utility in research and public health applications.

Through robust statistical analyses, the study aspires to position the REST Questionnaire as a valuable instrument for understanding the multifaceted impact of fatigue and for guiding targeted interventions in clinical and population health contexts.

### 2.4. Sample Size

This study required 250 participants, a sample size strategically determined to ensure statistical robustness and validity in evaluating fatigue and insomnia scores. This size exceeds the minimum threshold of 200 participants, which is recommended for reliability analyses to produce stable estimates of Cronbach’s alpha and to minimize standard errors [7]. Moreover, it provides sufficient power to detect moderate correlations (r = 0.3) between fatigue and sleep scores, as well as other key health indicators, including WHO-5 well-being scores, BMI, and demographic variables. Based on Cohen’s power calculation guidelines, detecting such correlations at α = 0.05 with 80% power requires a minimum of 84 participants [8].

To enhance the study’s rigor, the sample size was also designed to support subgroup analyses across diverse demographic, socioeconomic, and health history groups. This enabled a detailed exploration of variations in fatigue and insomnia. In this study, the theoretical population was defined as adults aged 18 or older from the general population who were not under active treatment for major illnesses, ensuring that our findings reflect a community-based sample. Participants were recruited through an anonymous online survey distributed by collaborating healthcare professionals and administered using Google Forms, allowing for broad geographic reach and a diverse range of respondents. Accounting for a 10% incomplete response rate, the sample size ensures at least 225 valid responses, which is sufficient for conducting exploratory factor analysis (EFA), reliability testing, and external validity assessments. These design considerations align with established recommendations for psychometric studies [9,10].

### 2.5. Statistical Methods

#### 2.5.1. Descriptive Statistics

A comprehensive statistical methodology was employed to evaluate the psychometric properties of the fatigue scores, ensuring the reliability, validity, and interpretability of the results. Fatigue scores derived from the REST Questionnaire were calculated by summing the responses to the 13 individual items, each rated on a scale from 0 (“Not at all”) to 4 (“Very much”), with higher scores indicating greater fatigue severity. The WHO-5 Well-Being Index scores were rescaled to a 0–100 scale by summing the raw responses, dividing the total by the maximum possible score, and then multiplying the result by 100.

Descriptive statistics were used to summarize participant characteristics and variables. Continuous variables, such as self-reported height (in centimeters), were described using means, standard deviations, and ranges. Fatigue scores and WHO-5 scores were summarized with means, medians, interquartile ranges (IQRs), and standard deviations. Categorical variables, including gender, age groups (18–29, 30–49, 50–64, ≥65), and self-reported diseases (from the anamnesis checklist in Appendix B), were reported as frequencies and percentages.

Statistical analyses were performed in R version 4.4.2 using additional libraries as needed.

#### 2.5.2. Internal Consistency Analysis

To ensure the reliability of fatigue and insomnia scores, internal consistency was evaluated using Cronbach’s alpha, with a standard threshold of α > 0.7 indicating strong consistency [7]. The analysis involved assessing the contribution of each item to the overall scale, where items with corrected item–total correlations below 0.2 were flagged for potential revision. Subdomains of fatigue were examined individually to evaluate their reliability, ensuring that each dimension contributed meaningfully to the overall construct. Additionally, a sensitivity analysis was conducted to determine the impact of removing individual items on Cronbach’s alpha, balancing the need to maintain reliability with the preservation of the breadth of constructs measured by the scale. This comprehensive approach ensured the robustness of the instrument while maintaining its multidimensional integrity.

As part of a pre-planned exploratory analysis, a subgroup comparison was devised to evaluate the internal consistency of the fatigue scale between individuals with cancer and those without cancer. This analysis aimed to determine whether the reliability of the scale differed between these groups, given the unique characteristics of cancer-related fatigue. Cronbach’s alpha was chosen as the primary metric to assess internal consistency, with a minimum sample size threshold of 30 participants per group established to ensure stable reliability estimates. The analysis included a sensitivity check, removing items contributing weakly to the overall scale, and examined differences in item performance between the groups. This approach was designed to explore potential areas for refinement in the scale, particularly for its use in clinical populations.

#### 2.5.3. Correlation Analysis

The construct validity of fatigue scores was assessed by analyzing their relationships with external variables, including WHO-5 well-being scores, BMI, self-rated health, and the presence of chronic medical conditions. We hypothesized that REST scores would exhibit a negative correlation with WHO-5 well-being scores, reflecting an association between higher fatigue levels and lower well-being. Similarly, a positive correlation was expected between REST scores and BMI, indicating that individuals with a higher BMI might experience greater fatigue. We also anticipated a negative correlation between REST scores and self-rated health, suggesting that increased fatigue would correspond to poorer self-perceived health. Additionally, we hypothesized that individuals with chronic medical conditions would report higher REST scores compared to those without such conditions, aligning with established evidence linking chronic illness to elevated fatigue. These hypotheses were grounded in existing research findings [11,12,13,14,15] and theoretical considerations, supporting the use of these external variables to validate the REST Questionnaire.

Pearson’s correlation was utilized for continuous variables such as WHO-5 scores and BMI, assuming linear relationships and normality, while Spearman’s rank correlation was applied to ordinal or non-normally distributed variables, such as age groups and education levels. Subgroup comparisons were designed to assess differences in fatigue scores across demographic and health-related groups. For comparisons involving three or more groups, one-way ANOVA with post hoc tests was performed if assumptions of the normality and homogeneity of variances were met, while for two-group comparisons, a Student’s t-test was used under similar conditions. If these assumptions were violated, non-parametric alternatives, including the Kruskal–Wallis test for multiple groups or the Mann–Whitney U test for two groups, were employed to ensure the robustness and accuracy of the analyses.

#### 2.5.4. Principal Component Analysis (PCA)

To explore the underlying structure of the 13 fatigue-related items in the REST Questionnaire, principal component analysis (PCA) was conducted as an exploratory technique without a priori theoretical constructs. PCA was chosen over exploratory factor analysis (EFA) due to the lack of a strongly established theoretical framework underlying the questionnaire. This made PCA more suitable as it emphasizes data reduction and the identification of patterns in variance rather than hypothesizing latent constructs. PCA was also selected for its straightforward and robust approach to dimensional analysis, particularly when the goal is to summarize the data and identify key components for further investigation. Before PCA was conducted, the data were evaluated for suitability through standard preliminary checks, including the Kaiser–Meyer–Olkin (KMO) test for sampling adequacy and Bartlett’s test of sphericity to ensure sufficient correlations among items. PCA was then performed to extract components, with the number of components retained determined empirically using the Kaiser criterion (eigenvalues > 1) and supported by an examination of the scree plot. To enhance interpretability, Varimax rotation was applied only if more than one component was identified. This rotation method simplified the structure by maximizing the variance in squared loadings across components, providing a clearer representation of item associations. Items with loadings above 0.4 were considered significantly associated with a component, while items with cross-loadings exceeding 0.3 were flagged for potential review. The explained variance for each component and the cumulative variance were calculated.

#### 2.5.5. Categorization of Fatigue

Fatigue scores were categorized into ordinal levels to capture the variability in fatigue within the general population, recognizing that the scores might not adhere to a normal distribution. To accommodate this, non-parametric methods, including the Kruskal–Wallis H test, were identified as appropriate for comparing distributions across multiple groups. Additionally, the analysis framework incorporated the calculation of prevalence rates for categorical variables, enabling the assessment of trends and associations between fatigue levels and various demographic and health-related factors.

## 3. Results

### 3.1. Descriptive Statistics

Among the participants, 159 were women and 109 were men. The age distribution showed that the largest group (63 individuals) was in the 26–30 age range, with smaller numbers in other ranges, including only 3 participants over 80. The provinces of residence indicated that most participants lived in Napoli (126) and Salerno (100), with fewer in Roma (14), Milano (8), and Caserta (6), and a collective category of other provinces with fewer than 5 individuals each was formed. Educational attainment was dominated by those with a university degree (162 participants), followed by high school graduates (73), middle school certificate holders (25), elementary certificate holders (6), and those with no formal education (2).

Key variable statistics revealed that the WHO-5 Well-Being Index had a mean score of 54.61, a standard deviation of 18.12, a median of 56.00, and an interquartile range (IQR) of 44–68, while BMI averaged 26.68 with a standard deviation of 5.74, a median of 25.59, and an IQR of 21.86–27.37. Fatigue scores averaged 14.04 with a standard deviation of 10.24, a median of 12.00, and an IQR of 6–20. For the self-assessment with overall general health ratings, the mean was 3.89, with a standard deviation of 0.86.

Frequency tables detail individual responses to fatigue questions.

Finally, data on reported diseases indicated that 146 participants reported no health conditions, while others noted specific issues, including high cholesterol or triglycerides (73), high blood pressure (59), cancer (33), kidney stones (19), autoimmune diseases (13), diabetes (9), and intestinal polyps (7), with smaller numbers of participants reporting other conditions such as strokes, gallstones, and angina pectoris. These results collectively offer a detailed view of the study population’s characteristics and health outcomes.

See Table 1, Table 2, Table 3 and Table 4 for more details.

### 3.2. Internal Consistency

#### 3.2.1. Overall Population

The analysis of the fatigue scale revealed an overall Cronbach’s alpha of 0.918, demonstrating excellent internal consistency across all items. Corrected item–total correlations were also examined, with none falling below the critical threshold of 0.2, indicating that all items contribute adequately to the scale’s reliability. The lowest corrected item–total correlation was observed for Question 3 (“How frequently have you experienced headaches in the last 30 days?”) with a correlation of 0.395, while high-contributing items included Question 8 (“How much has fatigue affected your social life?”) with a correlation of 0.791, Question 6 (“How much has fatigue limited your ability to plan the activities you want to do?”) with a correlation of 0.780, and Question 13 (“How often have you felt the need to seek medical help because of fatigue?”) with a correlation of 0.770. The sensitivity analysis showed that removing Question 3 increased Cronbach’s alpha to 0.922, suggesting that it is the weakest contributor to the scale’s overall reliability, while removing other items generally reduced reliability, with notable drops when high-contributing items like Question 8 were excluded (alpha reduced to 0.906).

#### 3.2.2. Populations with and Without Cancer

The subgroup analysis included 33 individuals with cancer and demonstrated a Cronbach’s alpha of 0.861 for the fatigue scale, indicating strong internal consistency. In contrast, the group without cancer showed a higher Cronbach’s alpha of 0.926, reflecting excellent reliability. In the group with cancer, Item 3 (“How frequently have you experienced headaches in the last 30 days?”) and Item 10 (“How often have you felt the need to consume coffee, tea, energy drinks, or supplements to combat fatigue?”) were identified as weaker contributors to scale reliability, with corrected item–total correlations of 0.413 and 0.217, respectively. Removing these items increased alpha slightly to 0.865, suggesting a potential improvement in consistency. Overall, the fatigue scale showed robust reliability in both groups, though minor item-level refinements may enhance its applicability in populations with cancer.

### 3.3. Correlation Analysis

Spearman’s correlation analysis was conducted to explore relationships between fatigue scores, WHO-5 well-being scores, self-judged health ratings, and BMI. The analysis revealed a moderate negative correlation between fatigue and the WHO-5 Well-Being Index (r = −0.45), indicating that higher well-being scores are associated with lower fatigue levels. A weak negative correlation was observed between fatigue and self-rated overall health status (r = −0.29), suggesting that individuals with better self-rated health report lower fatigue. No significant relationship was found between fatigue and BMI (r = 0.04), implying that body mass index does not substantially influence fatigue in this dataset.

To further investigate fatigue’s associations with demographic variables and reported diseases, Pearson’s correlation was employed for continuous and binary variables. Significant positive correlations were found between fatigue and diabetes (r ≈ 0.13, *p* < 0.05), kidney stones (r ≈ 0.17, *p* < 0.05), cancer (r ≈ 0.15, *p* < 0.05), and gastric ulcers (r ≈ 0.18, *p* < 0.05). These findings suggest that individuals with these conditions experience higher fatigue levels, likely due to the physiological, emotional, or systemic impacts of these diseases. Additionally, gender exhibited a weak negative correlation (r ≈ −0.23, *p* < 0.05), with women reporting higher fatigue scores than men. Conversely, variables such as education (r ≈ 0.09), cholesterol or triglycerides (r ≈ −0.01), high blood pressure (r ≈ −0.02), gallstones (r ≈ 0.04), and intestinal polyps (r ≈ −0.01) did not show statistically significant associations with fatigue, suggesting limited or negligible influence.

Subgroup analyses were performed using non-parametric methods due to the non-normal distribution of fatigue scores. The Mann–Whitney U test revealed significant differences in fatigue levels by sex (U = 11,111.0, *p* = 0.0001), highlighting gender as an important factor. Significant differences were also observed for cancer (U = 5034.0, *p* = 0.0055) and kidney stones (U = 1662.5, *p* = 0.0308), indicating that these conditions are associated with higher fatigue. Other binary variables, such as stroke (U = 38.0, *p* = 0.2191), diabetes (U = 820.5, *p* = 0.1314), and high cholesterol (U = 7424.5, *p* = 0.5871), did not demonstrate significant differences in fatigue scores. Non-significant findings were also observed for autoimmune diseases (U = 1867.5, *p* = 0.4418), gallstones (U = 523.5, *p* = 0.4364), and intestinal polyps (U = 914.5, *p* = 0.9980).

For categorical variables, the Kruskal–Wallis H test found no significant differences in fatigue scores across age groups (H = 4.91, *p* = 0.9611) or educational attainment levels (H = 6.77, *p* = 0.1485), indicating that neither age nor education substantially influences fatigue within this dataset. Overall, these results underscore the complex interplay of demographic and health factors in determining fatigue, with certain conditions and gender playing more pronounced roles.

### 3.4. Multivariable Linear Regression

To address multicollinearity concerns, a series of tests were conducted to validate the assumptions required for applying multilinear regression. Linearity was confirmed through the residuals versus predicted values plot, which showed no discernible patterns, indicating a linear relationship between the predictors and the response variable. Homoscedasticity was assessed by examining the spread of residuals across predicted values, which appeared relatively consistent, suggesting that the assumption of constant variance was reasonably met. The normality of residuals was evaluated using a histogram, which displayed a roughly symmetric distribution, though the Shapiro–Wilk test (*p* = 1.87 × 10^−6^) indicated some deviation from normality. While this departure could influence inference, the effect is mitigated by the large sample size, which stabilizes model performance.

Multicollinearity was addressed through Variance Inflation Factor (VIF) analysis, where all predictors showed VIF values below the critical threshold of 10. This was achieved by excluding high-VIF predictors, such as self-judged health and province of residence, ensuring the model’s stability and reducing redundancy. Based on these assessments, the assumptions for multilinear regression were largely satisfied, making the model suitable for analysis and inference.

The results of the regression analysis identified significant predictors of fatigue levels. WHO-5 well-being scores had the strongest relative impact, with a standardized coefficient of −0.3906 (*p* < 0.0001), indicating that higher well-being significantly reduced fatigue. Gastric ulcers (standardized coefficient = 0.1871, *p* = 0.0005) and cancer (standardized coefficient = 0.1911, *p* = 0.0037) were also significant positive predictors, reflecting their strong associations with increased fatigue. Gender showed a negative standardized coefficient of −0.1805 (*p* = 0.0013), suggesting that men reported lower fatigue levels compared to women. Kidney stones were another significant predictor (standardized coefficient = 0.1275, *p* = 0.0337), indicating their influence on fatigue levels.

Other predictors, including age (standardized coefficient = −0.1307, *p* = 0.0972), cholesterol/triglycerides (standardized coefficient = 0.0956, *p* = 0.2113), and diabetes (standardized coefficient = 0.0702, *p* = 0.2534), did not reach statistical significance. Similarly, BMI, educational level, and a history of infarction, gallstones, angina, high blood pressure, stroke, autoimmune diseases, or intestinal polyps showed minimal or negligible impacts, as indicated by their non-significant *p*-values and low standardized coefficients.

Overall, the model successfully identified key predictors of fatigue, emphasizing the roles of well-being, gastric ulcers, cancer, gender, and kidney stones. The satisfaction of most regression assumptions supports the reliability and interpretability of these findings, which highlight the complex interplay of health and demographic factors in determining fatigue levels.

See Table 5 for more details.

### 3.5. Principal Component Analysis

PCA was conducted using the principal component method, with Varimax rotation applied to enhance interpretability after identifying more than one component. Preliminary tests confirmed the data’s suitability for dimensional analysis, with a Kaiser–Meyer–Olkin (KMO) measure of 0.91 indicating excellent sampling adequacy and Bartlett’s test of sphericity yielding a highly significant result (χ^2^ = 2181.50, *p* < 0.001), confirming sufficient inter-item correlations. An analysis of eigenvalues and the scree plot identified two distinct components with eigenvalues greater than 1, together explaining 61.25% of the total variance. The first component, which explained 42.84% of the variance, primarily captured the emotional and social consequences of fatigue, while the second component, accounting for an additional 18.40%, reflected physical fatigue and its functional impacts. Varimax rotation revealed strong item loadings (≥0.4) on their respective components, with no problematic cross-loadings or items requiring revision, underscoring a clear and interpretable two-dimensional structure of fatigue-related items in the REST Survey.

Details of the explained variance and item–component associations are presented in Table 6 and Table 7.

### 3.6. Categorization of Fatigue

Fatigue scores, ranging from 0 to 52, were categorized into three levels—low (≤20), medium (21–35), and high (≥36)—reflecting the expected natural distribution within a general population sample. These thresholds effectively differentiated fatigue levels in relation to general well-being and health metrics. The Kruskal–Wallis H test showed significant differentiation in well-being scores and self-rated health, with both metrics decreasing as fatigue levels increased, while BMI showed no significant variation across fatigue levels. Diabetes prevalence increased from 1.99% in the low-fatigue group to 7.02% in the medium-fatigue group and 10.00% in the high-fatigue group. Similarly, the prevalence of kidney stones rose progressively from 5.47% in the low-fatigue group to 8.77% in the medium-fatigue group and 30.00% in the high-fatigue group. Gastric ulcers, nearly absent in the low- (0.50%) and medium-fatigue (0.00%) groups, rose dramatically in prevalence to 20.00% in the high-fatigue group. Cancer prevalence showed a nuanced pattern, peaking at 22.81% in the medium-fatigue group compared to 9.45% and 10.00% in the low- and high-fatigue groups, respectively. High blood pressure was moderately represented across all fatigue levels, peaking slightly in the medium-fatigue category but lacking a clear trend, while intestinal polyps were minimally represented and evenly distributed. These results confirm that the proposed thresholds for fatigue levels effectively differentiate key metrics and highlight meaningful associations between fatigue and chronic health conditions.

## 4. Discussion

This study provides preliminary evidence supporting the REST Questionnaire as a potential tool for assessing fatigue in the general population. The results suggest that the REST Questionnaire demonstrates good internal consistency (Cronbach’s alpha = 0.918) and appears to show strong construct validity, with notable correlations with well-being and self-rated health. Higher fatigue scores were associated with lower well-being, female gender, and the presence of chronic conditions such as cancer, kidney stones, and gastric ulcers. The REST Questionnaire represents a significant advancement in fatigue assessment, addressing the limitations of existing tools like the Fatigue Severity Scale (FSS) and the Chalder Fatigue Scale (CFS). While the FSS focuses primarily on functional impairment and the CFS distinguishes between physical and mental fatigue, the REST Questionnaire adopts a holistic approach. It integrates physical, emotional, and functional dimensions, including anticipatory limitations, emotional responses, and coping mechanisms, into a single, concise measure. This comprehensive scope positions the REST Questionnaire as a valuable tool for both clinical and research applications, surpassing the narrower focus of its predecessors. One of the primary advantages of the REST Questionnaire lies in its ability to integrate physical, emotional, and functional domains of fatigue into a single, concise measure. Furthermore, the FSS and CFS have been instrumental in clinical research but are often limited in scope. For example, the FSS focuses heavily on the physical manifestations of fatigue, particularly in chronic conditions like multiple sclerosis and lupus [16]. While it offers strong internal consistency and validity for assessing fatigue in these contexts, its applicability to the general population may be constrained by its narrow focus. Similarly, the CFS, though useful for measuring fatigue severity and its psychological impact, has faced criticism for its limited sensitivity to lifestyle factors and its applicability across diverse populations [3].

One feature of practical interest of the REST Questionnaire is its concise yet comprehensive design. Tools like the Multidimensional Fatigue Inventory (MFI) provide valuable insights into different dimensions of fatigue but are often lengthy and cumbersome, limiting their utility in large-scale studies [17]. The REST Questionnaire’s brevity ensures ease of administration while preserving the depth needed to capture the multifaceted nature of fatigue. This balance of precision and practicality is critical in public health research, where the ability to efficiently gather data from large and diverse populations is paramount.

The findings from this study highlight the REST Questionnaire’s strong internal consistency, as evidenced by its Cronbach’s alpha of 0.918, which exceeds the standard threshold for reliability. This high level of internal consistency suggests that the questionnaire items are well correlated and effectively capture a cohesive construct of fatigue. Exploratory factor analysis (EFA) further supported its validity, revealing two distinct dimensions of fatigue: physical–functional and emotional–social. These findings align with theoretical models of fatigue that emphasize its multidimensional nature [18]. The identification of these dimensions not only enhances the interpretability of REST scores but also facilitates targeted interventions aimed at specific aspects of fatigue.

The REST Questionnaire also stands out for its ability to differentiate fatigue levels across health and demographic subgroups. Our findings, which show a significant association between increased fatigue, female gender, and lower well-being scores, are consistent with existing research. A recent meta-analysis [19] identified female gender as a significant factor associated with increased fatigue in the general population, while other studies have established a link between higher fatigue levels and lower levels of general well-being [12,20]. In our study, significant associations were also observed between fatigue scores and chronic health conditions such as cancer, kidney stones, and gastric ulcers. These findings align with previous research linking these conditions to fatigue. In one study involving 385 patients with peptic ulcers, 50–60% reported that their physical activity was limited in some way [21]. A study of 103 patients with urolithiasis demonstrated significantly reduced physical function and increased pain levels compared to the general population [22]. A meta-analysis of 129 studies (71,568 patients with cancer) found an overall fatigue prevalence of 49% (95% CI = 45–53%), with rates highest in patients with advanced cancer (60.6%) and those undergoing treatment (62%). Fatigue prevalence declined over time, from 64% in studies from 1996–2000 to 43% in 2016–2020, with female gender identified as a significant predictor of higher fatigue rates [13].

Although our study included a subgroup of individuals with a history of cancer (n = 33), it is essential to note that these participants were cancer-free and not undergoing active treatment. This distinction is critical because the nature and underlying mechanisms of fatigue may differ between individuals currently battling cancer and those who are in remission. Cancer-related fatigue (CRF) is a complex phenomenon often influenced by factors such as ongoing inflammation, treatment side effects, and psychological distress, which are not explicitly addressed by the REST Questionnaire.

Within the group with cancer, two items from the REST fatigue scale were identified as weaker contributors to reliability. Item 3 (“How frequently have you experienced headaches in the last 30 days?”) and Item 10 (“How often have you felt the need to consume coffee, tea, energy drinks, or supplements to combat fatigue?”) showed corrected item–total correlations of 0.413 and 0.217, respectively. Removing these items increased the Cronbach’s alpha for the REST fatigue scale slightly to 0.865, suggesting that minor refinements could further enhance the scale’s internal consistency for populations with cancer.

It is important to recognize that cancer-related fatigue often arises from complex, multifactorial causes, including treatment side effects, chronic inflammation, and emotional distress, which may influence responses differently compared to in the general population [23]. For example, headaches in patients with cancer might be secondary to specific factors like chemotherapy, dehydration, or stress, rather than directly linked to fatigue itself. This variability might reduce the item’s specificity and overall contribution to assessing fatigue. Similarly, the “use of stimulants” item may not accurately reflect fatigue severity in patients with cancer, as stimulant consumption in this group could stem from diverse motivations. For instance, patients may consume caffeine or supplements to manage treatment side effects like nausea or to maintain energy during demanding treatment regimens, irrespective of their fatigue levels. These contextual nuances may dilute the item’s relevance to fatigue in this specific subgroup. Given the small size of the subgroup with cancer, caution is warranted in overgeneralizing these findings. However, these considerations suggest potential areas for refinement. Rephrasing the “headaches” item to specify fatigue-related headaches or clarifying the “stimulant use” item to differentiate between fatigue mitigation and other reasons for use could enhance the tool’s construct validity in clinical populations. Although our preliminary results show that the REST fatigue scale demonstrated robust reliability in patients with a history of cancer, future studies focusing specifically on populations with cancer, with larger and more diverse samples, are needed to validate it in patients of this category and further explore subgroup-specific variations in fatigue assessment.

The FACIT-Fatigue scale, in contrast, was explicitly designed to assess fatigue in patients with cancer, including those undergoing treatment and survivors. It builds upon the Functional Assessment of Cancer Therapy—General (FACT-G) questionnaire by adding 13 fatigue-specific items, making it a powerful tool for evaluating fatigue in oncology populations. The FACIT-Fatigue scale has demonstrated strong psychometric properties, with internal consistency values (Cronbach’s alpha) ranging from 0.93 to 0.95 [24], and has also been employed to explore the effectiveness of specific interventions against cancer-associated fatigue [25]. Its validation studies indicate strong test–retest reliability (r = 0.87) and the ability to differentiate between patients based on hemoglobin levels and performance status, highlighting its relevance in both clinical and research settings. Despite its strengths, the FACIT-Fatigue scale has notable limitations when applied to cancer survivors not currently undergoing treatment. While it effectively measures treatment-related fatigue and anemia-associated concerns, it may not fully capture anticipatory, social, or emotional aspects of fatigue commonly experienced by long-term survivors. The REST fatigue scale, in comparison, offers a broader multidimensional approach that includes the social and emotional impacts of fatigue, providing a complementary perspective to the FACIT-Fatigue scale.

An exploratory factor analysis of the REST Survey’s fatigue-related items revealed a two-factor structure, accounting for 61.25% of the total variance. These factors, identified as “physical fatigue and functional impacts” and “emotional and social consequences of fatigue”, underscore the multidimensional nature of fatigue captured by the instrument. This factor structure highlights the survey’s capacity to provide a nuanced assessment of fatigue, supporting its diagnostic and evaluative utility for both research and intervention development. The categorization of fatigue scores into low, medium, and high levels further demonstrated the REST Questionnaire’s utility in differentiating key health outcomes. Participants with higher fatigue levels exhibited lower well-being scores, as measured by WHO-5, and an increased prevalence of chronic conditions such as diabetes and gastric ulcers. These findings align with studies linking higher fatigue levels to poorer health outcomes and reduced quality of life [19]. The REST tool’s ability to stratify individuals based on fatigue severity provides valuable insights for both research and public health applications, enabling the identification of high-risk groups and the tailoring of interventions to their specific needs.

While the REST Questionnaire offers several advantages, it is important to acknowledge its limitations. The cross-sectional design of this study limits the ability to draw causal inferences regarding the relationships between fatigue and external variables. Longitudinal research is needed to establish the directionality of these associations and to explore the REST tool’s responsiveness to interventions aimed at reducing fatigue. Additionally, while the sample size was sufficient for psychometric analyses, larger and more diverse samples would enhance the generalizability of the findings and allow for more detailed subgroup analyses. Furthermore, while principal component analysis (PCA) was employed to explore the underlying structure of the REST Questionnaire, it is important to acknowledge that parallel analysis was not conducted. Parallel analysis could have provided a more robust method for determining the number of components to retain. However, due to the exploratory nature of this aspect of the study and the focus on initial validation, we relied on the Kaiser criterion and scree plot examination. Future studies should consider incorporating parallel analysis to further validate the factor structure of the REST Questionnaire. Finally, the lack of a significant association between fatigue scores and BMI in this study contrasts with some previous findings suggesting a link between obesity and fatigue [14]. This discrepancy may be explained by the REST Questionnaire’s holistic design, which emphasizes the multidimensional nature of fatigue, including emotional and social factors. Unlike physical fatigue, which may be more directly influenced by BMI, the emotional and functional dimensions assessed by the REST Questionnaire might dilute the apparent influence of BMI on overall fatigue scores. Additionally, our sample consisted predominantly of participants from a general population rather than from clinical populations where obesity and associated comorbidities are more prevalent.

It is also possible that confounding factors, such as physical activity levels, sleep quality, or psychological stress, mediate the relationship between BMI and fatigue, obscuring a direct correlation. These findings underscore the importance of considering fatigue as a multidimensional construct and suggest that future studies should include more comprehensive measures of physical and lifestyle factors to disentangle the complex interactions influencing fatigue. Longitudinal research examining the temporal relationships between BMI, lifestyle behaviors, and fatigue dimensions could further clarify these dynamics. The REST Questionnaire’s ability to disentangle the physical, emotional, and functional dimensions of fatigue offers a more nuanced understanding of these relationships, underscoring its value in capturing the complexity of fatigue as a construct.

Future research should also explore the REST Questionnaire’s applicability in specific populations, such as individuals with chronic illnesses or occupational fatigue. The tool’s holistic framework and emphasis on lifestyle factors make it particularly well suited for these contexts, where fatigue often arises from a complex interplay of physical, emotional, and environmental factors. Furthermore, adapting the REST tool for use in clinical settings could provide valuable insights into the impact of medical treatments on fatigue levels and inform strategies for improving patient outcomes.

In conclusion, the REST Questionnaire may represent a valuable advancement in the assessment of fatigue, addressing the limitations of existing tools and offering a comprehensive yet practical solution for research and public health applications. Its satisfactory psychometric properties, multidimensional framework, and sensitivity to comorbidities position it as a valuable instrument for understanding and addressing fatigue in diverse populations. By providing a nuanced perspective on fatigue and its associations with health and comorbid conditions, the REST Questionnaire has the potential to advance fatigue research and inform targeted interventions aimed at improving well-being, both in the general population and in patients with a history of cancer.

## Figures and Tables

**Table 1:** Counts for each categorical variable (combined count and percentage).

Variable	Category	Count (Percentage)
Sex	Women	159 (59.33%)
	Men	109 (40.67%)
Age Range	18–25	13 (4.85%)
	26–30	63 (23.51%)
	31–35	33 (12.31%)
	36–40	24 (8.96%)
	41–45	28 (10.45%)
	46–50	23 (8.58%)
	51–55	21 (7.84%)
	56–60	19 (7.09%)
	61–65	18 (6.72%)
	66–70	8 (2.99%)
	71–75	9 (3.36%)
	76–80	6 (2.24%)
	Over 80	3 (1.12%)
Province of Residence	Napoli	126 (47.01%)
	Salerno	100 (37.31%)
	Roma	14 (5.22%)
	Milano	8 (2.99%)
	Caserta	6 (2.24%)
	Others (<5 each)	14 (5.22%)
Education Level	University Degree	162 (60.45%)
	High School Diploma	73 (27.24%)
	Middle School Certificate	25 (9.33%)
	Elementary Certificate	6 (2.24%)
	None	2 (0.75%)

**Table 2:** Descriptive statistics of key variables.

Variable	Mean	Std Dev	Median	IQR
WHO-5 Well-Being Index (0–100)	54.61	18.12	56.00	44–68
BMI	26.68	5.74	25.59	21.86–27.37
Self-rated health score	3.88	0.86	4.0	3–4
REST score	14.04	10.24	12.0	6–20

**Table 3:** Frequency table for the REST Questionnaire.

Answers: Questions	0 = Not at All	1 = A Little	2 = Moderately	3 = A Lot	4 = Very Much
How often have you felt tired, fatigued, without energy, or exhausted?	21 (7.84%)	92 (34.33%)	86 (32.09%)	51 (19.03%)	18 (6.72%)
How often have you experienced muscle pain?	69 (25.75%)	94 (35.07%)	61 (22.76%)	33 (12.31%)	11 (4.10%)
How often have you suffered from headaches?	99 (36.94%)	79 (29.48%)	53 (19.78%)	26 (9.70%)	11 (4.10%)
How often have you felt as if your bones were aching or broken?	173 (64.55%)	40 (14.93%)	31 (11.57%)	15 (5.60%)	9 (3.36%)
How much has fatigue prevented you from performing your usual activities (work, study, household chores, etc.)?	122 (45.52%)	83 (30.97%)	41 (15.30%)	14 (5.22%)	8 (2.99%)
How much has fatigue limited your ability to plan the activities you want to do?	108 (40.30%)	83 (30.97%)	48 (17.91%)	20 (7.46%)	9 (3.36%)
How much has fatigue affected your work or study life?	109 (40.67%)	95 (35.45%)	35 (13.06%)	23 (8.58%)	6 (2.24%)
How much has fatigue affected your social life?	97 (36.19%)	83 (30.97%)	55 (20.52%)	23 (8.58%)	10 (3.73%)
How much has fatigue affected your emotional life and intimate relationships?	105 (39.18%)	83 (30.97%)	53 (19.78%)	16 (5.97%)	11 (4.10%)
How frustrated have you felt because of your fatigue?	67 (25.00%)	93 (34.70%)	63 (23.51%)	31 (11.57%)	14 (5.22%)
How worried have you felt because of your fatigue?	192 (71.64%)	38 (14.18%)	23 (8.58%)	6 (2.24%)	9 (3.36%)
How often have you felt the need to consume coffee, tea, energy drinks, or supplements to combat fatigue?	118 (44.03%)	67 (25.00%)	38 (14.18%)	29 (10.82%)	16 (5.97%)
How often have you felt the need to seek medical help because of fatigue?	127 (47.39%)	76 (28.36%)	22 (8.21%)	23 (8.58%)	20 (7.46%)

**Table 4:** Self-reported medical history.

Medical History	Count (Percentage)
None of the conditions listed	146 (38.83%)
Elevated cholesterol or triglycerides	73 (19.41%)
Hypertension (High blood pressure)	59 (15.69%)
Cancer	33 (8.78%)
Renal calculi (Kidney stones)	19 (5.05%)
Autoimmune diseases	13 (3.46%)
Diabetes	9 (2.39%)
Intestinal polyps	7 (1.86%)
Myocardial infarction	5 (1.33%)
Liver stones (Gallstones)	5 (1.33%)
Gastric or duodenal ulcer	3 (0.8%)
Angina pectoris	3 (0.8%)
Cerebral stroke	1 (0.27%)

**Table 5:** Regression coefficients and significance.

Feature	*p*-Value	Standardized Coefficient
WHO100	<0.0001	−0.3906
Gastric or duodenal ulcer	0.0005	0.1871
Gender	0.0013	−0.1805
Cancer	0.0037	0.1911
Renal calculi (Kidney stones)	0.0337	0.1275
Age (18 to over 80 years)	0.0972	−0.1307
Elevated cholesterol or triglycerides	0.2113	0.0956
Diabetes	0.2534	0.0702
Body mass index	0.3450	0.0512
Myocardial infarction	0.3509	0.0619
Liver stones (Gallstones)	0.5654	0.0348
Angina pectoris	0.6027	0.0337
Hypertension (High blood pressure)	0.7777	−0.0203
Educational qualification	0.8142	0.0135
Cerebral stroke	0.8726	−0.0100
Autoimmune diseases	0.8895	−0.0079
Intestinal polyps	0.9559	0.0031

**Table 6:** Variance explained by components.

Component	Eigenvalue	Variance Explained (%)	Cumulative Variance (%)
Component 1—Emotional and social consequences of fatigue	5.57	42.84	42.84
Component 2—Physical fatigue and its functional impacts	2.39	18.40	61.25

**Table 7:** Rotated component matrix (significant loadings ≥ 0.4): association between items and components.

Questions	Component 1—Emotional and Social Consequences of Fatigue	Component 2—Physical Fatigue and Its Functional Impacts
How often have you felt tired, fatigued, without energy, or exhausted?	0.55	0.44
How often have you experienced muscle pain?		0.75
How often have you suffered from headaches?		0.65
How often have you felt as if your bones were aching or broken?		0.81
How much has fatigue prevented you from performing your usual activities (work, study, household chores, etc.)?	0.75	
How much has fatigue limited your ability to plan the activities you want to do?	0.84	
How much has fatigue affected your work or study life?	0.76	
How much has fatigue affected your social life?	0.85	
How much has fatigue affected your emotional life and intimate relationships?	0.76	
How frustrated have you felt because of your fatigue?	0.51	
How worried have you felt because of your fatigue?	0.61	0.40
How often have you felt the need to consume coffee, tea, energy drinks, or supplements to combat fatigue?	0.80	
How often have you felt the need to seek medical help because of fatigue?	0.79	

## Data Availability

Data are unavailable due to privacy restrictions.

## Appendix A

The REST (Recognizing and Estimating Signs of Tiredness) Survey.

Please answer the following questions carefully. We remind you that your responses will be sent to you via email, and you can discuss them with your trusted doctor. The questions refer to your experiences over the last 30 days.

Please keep the following scale in mind while answering:

0 = Not at all

1 = A little

2 = Moderately

3 = A lot

4 = Very much

Questions:

How often have you felt tired, fatigued, without energy, or exhausted?

How often have you experienced muscle pain?

How often have you suffered from headaches?

How often have you felt as if your bones were aching or broken?

How much has fatigue prevented you from performing your usual activities (work, study, household chores, etc.)?

How much has fatigue limited your ability to plan the activities you want to do?

How much has fatigue affected your work or study life?

How much has fatigue affected your social life?

How much has fatigue affected your emotional life and intimate relationships?

How frustrated have you felt because of your fatigue?

How worried have you felt because of your fatigue?

How often have you felt the need to consume coffee, tea, energy drinks, or supplements to combat fatigue?

How often have you felt the need to seek medical help because of fatigue?

Calculation Methods Scoring: Each question will be scored on a scale from 0 (Not at all) to 4 (Very much). The total score is calculated by summing the individual responses for all 13 questions. The total score can range from 0 to 52, with higher scores indicating greater severity and impact of fatigue.

## Appendix B. List of Diagnosed Diseases in the Anamnesis Section

Participants were asked the following: “Has a doctor ever diagnosed you with the following conditions?” (Multiple responses were allowed).

Cardiovascular diseases: Myocardial infarction, angina pectoris, stroke, hypertension.

Metabolic disorders: High cholesterol or triglycerides, diabetes.

Gallstones or kidney stones.

Gastrointestinal conditions: Intestinal polyps, gastric or duodenal ulcers.

Autoimmune diseases.

Malignant neoplasms:

Unspecified neoplasms: “I have suffered from a malignant neoplasm but cannot specify which.”

Specific cancers:

Lung carcinoma

Breast carcinoma

Prostate carcinoma

Colorectal carcinoma

Gastric carcinoma

Pancreatic carcinoma

Hepatocellular carcinoma

Esophageal carcinoma

Bladder carcinoma

Renal cell carcinoma

Cervical carcinoma

Endometrial carcinoma

Ovarian carcinoma

Thyroid carcinoma

Melanoma

Basal cell carcinoma

Squamous cell carcinoma

Osteosarcoma, chondrosarcoma, sarcoma of Ewing, rhabdomyosarcoma, leiomyosarcoma, liposarcoma.

Brain tumors, neuroendocrine tumors, germinal tumors.

Hematological malignancies: Acute myeloid leukemia (AML), chronic myeloid leukemia (CML), acute lymphoblastic leukemia (ALL), chronic lymphocytic leukemia (CLL), Hodgkin’s lymphoma, non-Hodgkin’s lymphoma, multiple myeloma.
